# Unanswered Questions in Clinical Practice

**DOI:** 10.1002/mdc3.13837

**Published:** 2023-08-04

**Authors:** Tim Lynch, Oscar S Gershanik

**Affiliations:** ^1^ Dublin Neurological Institute, Mater Misericordiae University Hospital Dublin Ireland; ^2^ Health Affairs and School of Medicine and Medical Science, University College Dublin Dublin Ireland; ^3^ Institute of Neuroscience, Favaloro Foundation University Hospital Buenos Aires Argentina

**Keywords:** antibody testing, biomarkers, COVID 19, essential tremor, gastrointestinal tract, genetic testing, Movement disorders, off‐label prescribing, Parkinson's disease, tremor

We had the pleasure of chairing 1 day of the highly successful International Parkinson and Movement Disorders Society (MDS) *Movement Disorder Clinical Practice* conference in November 2021. There were many presentations about controversial topics by experts in movement disorders followed by vigorous discussion.

Day 1 of the conference started with a timely review of movement disorders and severe acute respiratory syndrome coronavirus 2 (SARS‐COV2) by Alfonso Fasano.^1^ Pre‐existing comorbidities influenced the outcome of infected people with Parkinson's disease (PWP) leading to increased hospitalization and a variable increase in mortality. Social isolation imposed by the prolonged lock‐down also contributed to the worsening of motor and non‐motor symptoms. Similar issues were seen in patients with Tourette syndrome. Special attention should be paid to the “long‐COVID syndrome,” not exclusive to Parkinson's disease (PD), that led to an increase in motor symptomatology, medication use, cognitive, and sleep disorders. The most challenging question raised by Dr. Fasano relates to the issue of new‐onset movement disorders. Myoclonus was the most commonly observed movement disorder. Does viral infection, through a direct brain effect, cause movement disorders or do other factors play a role? Apparently, immune mediated mechanisms, combined with metabolic, vascular, endothelial dysfunction leading to blood–brain barrier disruption, in combination with medication side‐effects, are responsible for the majority of cases. There is no evidence of direct viral penetration into the brain. Whether coronavirus disease 2019 (COVID 19) causes new‐onset parkinsonism is very unclear. During the discussion it was felt that the most plausible explanation is the unmasking of pre‐clinical PD by the infection. The final “pearl” of the conference dealt with the rapid‐onset of tic‐like phenomena observed during the pandemic, which have been attributed to functional, socially driven phenomena (Tic‐Toc‐Tics).

Dr. Brit Mollenhauer reviewed the role of biomarkers in clinical practice and divided them into markers for diagnosis (state), progression (rate), clinical course (fate), and risk (trait).^2^ This classification provides a useful framework to understand their value in the diagnosis of PD, the differential diagnosis of parkinsonism, and for evaluating prognosis and risk factors for disease development. Although there has been tremendous progress, except for disease course biomarkers, the remaining biomarkers do not yet distinguish the different synucleinopathies at the preclinical or early disease stage for an individual. The newly developed seeding aggregation assays for α‐synuclein will probably improve our accuracy in diagnosis and in differentiating synucleinopathies from other forms of degenerative parkinsonism when they become widely available. Furthermore, they may help in identifying distinct types of synucleinopathies through the analysis of their dynamic properties.

Dr. Anthony Lang spoke on off‐label prescribing of disease modifying therapies under investigation; one of the most controversial topics of this conference. Dr. Lang provided a very compelling and balanced approach to the problem. How does the clinician balance the urgency and desire of patients to get access to existing and approved medications for other disorders against the potential risks? Often these medications are proposed and promoted as beneficial for their movement disorder based on their mechanism of action elucidated experimentally or through big data analysis with artificial intelligence (AI) tools. The main principles to guide treating clinicians in such instances are the basic tenet of bioethics, “first do not harm” established by The Helsinki Declaration. In addition, we need to recognize that a drug proven safe and effective for one illness does not necessarily mean it will be safe and effective for a different patient population. Moreover, establishing or refuting the efficacy of a disease‐modifying agent in PD is extremely difficult because of the lack of sensitive biomarkers for disease progression, the need for long‐term studies, and the difficulty of applying large cohort data to an individual. We are often confronted by patients with a request for off‐label use of a medication claimed to be effective for their disease often promoted through informal channels of information (television, newspapers, and social media). The key is to educate our patients, to let them know about false claims and pseudoscience, and to reach a shared decision not just based on expectations, but on evidence.

Dr. Fiorella Contarino spoke on the role of gastrointestinal system in PD and approached this topic from four different perspectives, (1) known existence of constipation and gastroparesis and other gastrointestinal disorders in PD, and how they impact on the quality of life of PWP; (2) the more recent attribution of the gut as a possible starting site of neurodegeneration in PD, citing the “dual‐hit” hypothesis and the “body‐first/brain‐first” theories on the pathogenesis of the disease.^3^ These theories are based on the (i) finding of Lewy bodies in the intestinal wall; (ii) the possible role of altered intestinal permeability and local inflammation as drivers of neurodegeneration possibly spreading along the vagus nerve to the brainstem; (iii) the differential involvement of neural pathways through multimodal neuroimaging in PWP; (iv) reduced risk of developing PD in individuals post vagotomy; (v) and the altered composition of the microbiota in PD patients that could contribute to the pathogenesis. Furthermore, (3) gut microbiota are proposed as a possible trigger, of neurodegeneration and a possible factor in progression of pathology. Remarkably, gut microbiota can interfere with the levodopa benefit by reducing its bioavailability to the brain. Last, (4) the possibility of targeting microbiota to prevent disease onset or slow down disease progression or improve levodopa benefit through dietary modification (diet, probiotics, prebiotics, symbiotics, antibiotics) or fecal transplantation. Most of these hypotheses remain highly speculative, with the exception of the recommendation of healthy dietary habits (Mediterranean diet) for which there are substantial evidence of its benefit.

Dr. Christine Klein gave a talk on “Genetic Testing for GBA and LRRK2 mutations: Is it time for routine use?” Dr. Klein highlighted the many breakthroughs in the genetics of PD over the last 25 years.^4^ We are now entering the era of gene therapy, antisense oligonucleotide therapy, and neuroprotective therapies for PD. There are two groups to consider when contemplating routine genetic testing: PWP and people with a positive family history and at risk of developing PD. Should clinicians offer routine genetic testing for the more common casual gene variants, for instance, glucocerebrosidase (GBA) and leucine‐rich repeat kinase (LRRK2) pathogenic variants? In general, Dr. Klein suggested that the answer presently remains “No”. There is no United States Food and Drug Administration (FDA)‐approved gene‐targeted therapies in PD and genetic testing can have negative social, psychological, and economic implications in asymptomatic carriers. Furthermore, we are missing detailed widespread genotype–phenotype studies of patients with PD associated with different genetics variants to map out phenotypic heterogeneity (one gene with multiple phenotypes depending on the variant and other factors) and also genotypic heterogeneity (multiple genes resulting in a similar phenotype) in PD. However, genetic testing can be helpful when there are atypical features and uncertainty about the diagnosis, an earlier age‐at‐onset to help with prognosis, counseling of patients and family members about disease progression, disease development and reproductive decisions, patients’ interests, and recruitment into clinical trials especially for neuroprotective agents. Genetic testing can be expensive and often is not available to the majority of PWP. There are some research studies that offer free genetic testing to people with PD including the Parkinson's progression Markers Initiative sponsored by The Michael J. Fox Foundation and the PDGENEration (Parkinson's Disease Genetic registry) sponsored by the Parkinson’ Foundation (Video 1).

**Video 1:** Movement disorder clinical practice (MDCP) panel; Day 1, Panel 1 Recording.

Dr. Bettina Balint spoke on the “Relevance of Antibody Testing in Movement Disorders”. Neuroimmunology and movement disorders are a rapidly changing and developing field.^5^ Dr. Balint noted two patterns—neuronal antibody related movement disorders (NARMD) refers to antibody‐mediated movement disorders where the antibody is pathogenic or antibody‐associated movement disorders, where the antibodies act a biomarker but are not pathogenic. Distinct from neurogenetic disorders, the diagnosis of an autoimmune etiology immediately guides treatment and management (immunotherapy, screening for an underlying tumor or associated autoimmunity, and relapses) of the patient and lends urgency to an accurate diagnosis. The clinician should aim for a “molecular” diagnosis rather than a syndromic diagnosis based on a specific antibody that impact on treatment, prognosis, and monitoring. Dr. Balint highlighted the importance of clinical acumen to guide the appropriate antibody test(s) and of maintaining a high index of suspicion when dealing with a patient with a possible autoimmune movement disorder. Patients with atypical movement disorders (eg, N‐methyl‐D‐aspartate receptor [NMDAR], Gamma‐aminobutyric acid subtype A receptor [GABA_A_R], immunoglobulin to neuronal cell adhesion molecule 5 [IgLON5], or Contactin‐associated protein‐like 2 [CASPR2] are often misdiagnosed. Classically, we recognize that autoimmune encephalitides are often rapid in onset, but some can have a deceptively slow progressive course that mimic neurodegenerative disease (eg, NARMD associated with Leucine‐rich glioma‐inactivated [LGI1], Dipeptidyl‐Peptidase‐like Protein‐6 [DPPX], CASPR2, IgLON5, or seizure Related 6 Homolog Like 2 [SEZ6L2] mimicking progressive supranuclear palsy). Moreover, the diagnostic surrogate markers (magnetic resonance imaging and cerebrospinal fluid [CSF] analysis) sometimes are too insensitive and can be normal in autoimmune movement disorders, even in NARMD. Relying on imaging and CSF analysis alone will lead to patients with treatable autoimmune disease being undiagnosed. Similar to neurogenetics, antibody heterogeneity occurs where one autoimmune phenotype can be associated with multiple antibodies (eg, stiff person syndrome associated with anti‐glutamic acid decarboxylase [anti‐GAD] or glycine receptor, amphiphysin or DPPX) and phenotypic heterogeneity occurs where one antibody can be associated with multiple phenotypes (eg, anti‐GAD associated with stiff person syndrome or focal epilepsy or cerebellar ataxia or type 1 diabetes). Therefore, like neurogenetics, antibody panels are recommended and antibodies of unknown significance (AUS) exist similar to variants of unknown significance (VUS) when interpreting a genetic result. Clinicians need to be aware of the limitations and pitfalls of antibody testing. Paired serum and CSF samples increase specificity and sensitivity of testing and allows detection of intrathecal antibody production using the antibody index (CSF/serum difference of antibody amounts per weight unit immunoglobulin G; normal <1.5). If the antibody results are unexpectantly negative or unclear, good communication between the clinician and the laboratory research scientist is helpful as often using a different laboratory method (eg, tissue‐based immunohistochemistry to assess the staining pattern) as well as the standard cell‐based assay can sometimes provide the diagnosis (Video 2).

**Video 2:** Movement disorder clinical practice (MDCP) panel; Day 1, Panel 2 Recording.

Dr. Alberto Espay spoke on the pitfalls of the new tremor classification and gave a provocative critique of this new tremor classification proposed by the 2018 Task Force on Tremor of the International Parkinson and Movement Disorder Society.^6^ The task force adopted a two axis classification scheme used previously to classify dystonia in 2013—Axis 1 clinical classification and Axis 2 etiology. At the same time they preserved the term essential tremor (ET). Dr. Espay argued that there are seven pitfalls with this classification system. (1) The term “essential” is misleading; (2) ET is both an “isolated tremor” and an Axis 1 diagnosis. ET is defined as an “isolated tremor syndrome of bilateral upper limb action locations” for at least 3 years, with or without tremor in other locations and in the absence of neurological signs. (3) The 3‐year rule beyond which hand tremors must be essential is arbitrary and not based on any biological data. (4) ET can be essential, but have more than one etiology over time. However, it is understandable that sometimes our diagnoses change over time as new symptoms, signs, or tests results appear. (5) ET requiring an “isolated tremor” of action for at least 3 years implies the classification is made by exclusion. (6) Classification is a lumping approach to the diagnosis of tremor. (7) “ET plus” is based on lumping soft signs into ET and lacks scientific value. However, not uncommonly a clinician may be unsure of the diagnosis and describe what he or she sees and uses the term ET plus pending further follow up and assessment when the nature of the tremor evolves and the etiology becomes clear. Dr. Espay suggested clinicians should describe what they see rather than using the term ET or ET plus or indeterminate tremor. Dr. Espay also suggests ET should be used in Axis 2 rather than in Axis 1 if the term is to be retained. The panel discussion highlighted the fact that the 2018 classification is a work in progress and will evolve and change and that clinicians should describe what they see rather than rush to a label.

## Disclosures

**Funding Sources and Conflicts of Interest:** The authors have no conficts of interest. Dr Lynch has grant support from the Health Research Board Ireland, Michael J Fox Foundation and the Irish Institute of Clinical Neuroscience.

**Financial Disclosures for the Previous 12 Months:** Dr Gershanik has no financial disclosures. Dr Lynch received unrestricted educational grants from Bial, Biogen, Clonmel Healthcare, and Roche to support the Annual Parkinson's disease & other Movement Disorders and the Annual Neuroimmunolgy confernces at the Mater Misericordiae University Hospital, Dublin.
